# Complete mitochondrial genome of nearly threatened freshwater ornamental fish, *Microphis deocata* and its phylogenetic relationship within Syngnathidae

**DOI:** 10.1080/23802359.2020.1835572

**Published:** 2021-03-26

**Authors:** Lakshman Sahoo, Ashoktaru Barat, Paramananda Das, Bismay Sahoo, Gargee Das, Jitendra. K. Sundaray, Sangram K. Sahoo, Saroj K. Swain, Khuntia Murmu, Dandadhar Sarma

**Affiliations:** a Fish Genetics and Biotechnology Division, ICAR – Central Institute of Freshwater Aquaculture, Bhubaneswar, India; b Department of Zooology, Gauhati University, Guwahati, India

**Keywords:** Mitochondrial genome, Deocata, NGS, protein coding genes, phylogenetics, Syngnathidae

## Abstract

*Microphis deocata* (deocata pipefish), belonging to family Syngnathidae, is one of the important indigenous ornamental fish species listed as near threatened in the IUCN red list. Here, we first report the complete mitochondrial genome of deocata pipefish using Illumina next-generation sequencing platform. The total length of the mitogenome is 16,526 bp. It encompasses 13 protein coding genes, 2 ribosomal rRNAs, and 22 tRNAs. The WANCY region (a cluster of five tRNA genes) contains the 50 bp O_L_ light strand origin of replication. Phylogenetic analysis of Syngnathidae revealed *M. deocata* to cluster with *Oostethus manadensis,* forming a sister group with *Doryrhamphus japonicas* and *Dunckerocampus dactyliophorus.* The mitochondrial genome sequence data generated in the present study will play an important role in population genetic analysis and developing conservation strategies for this species.

## Introduction

*Microphis deocata,* popularly known as Indian royal green pipefish or rainbow belly pipefish or deocata pipefish, is a freshwater fish species belonging to the family Syngnathidae. The Syngnathids differ from other vertebrates by exhibiting ‘male pregnancy’ (Stölting and Wilson 2007). They have an extended body covered with armour of bony plates in place of scales, elongated snout and fused jaws (Wang et al. 2019). Due to specialized morphology, the fish is a popular aquarium fish with enormous demand. This has resulted in a decline in its population due to over exploitation. Now, the species is listed in the IUCN red list (https://www.iucnredlist.org/species/168512/67623942). Here, we first report the complete mitogenome of *M. deocata* and performed phylogenetic analysis among Syngnathidae species (27 species, 12 genera) available in the GenBank database. Fin sample of *M. deocata* (MD01) was collected from Narayanguri, Assam, India (GPS Coordinates: 90°59′43.2ʺE and 26°39′63.0ʺN) during September 2018 and kept in fish genomics laboratory of ICAR – Central Institute of Freshwater Aquaculture, Bhubaneswar, India. High molecular weight genomic DNA was isolated and the complete mitogenome of *M. deocata* was obtained by sequencing the DNA using Illumina Nextseq500 platform. A maximum likelihood (ML) tree based on the concatenated supergene consisting of 13 mitochondrial protein-coding genes was constructed using MEGAX.

The *M. deocata* mtDNA genome was 16,526 bp (Accession No. MT230531) in length and circular in shape. It contained 13 protein-coding genes (PCG), 22 tRNAs, 2 ribosomal RNAs and a putative control region. The arrangements of genes were identical to a typical vertebrate mitochondrial genome (Boore 1999; Bej et al. 2012: Sahoo et al. 2016). Like other vertebrate mitochondrial genome, the initiation codon for all 13 PCGs was ATG except *COI* where it was GTG. Most of the PCGs had TAA stop codon whereas ND2, COII, ND3, ND4, and Cytb had an incomplete stop codon T. In total, 11 intergenic regions with 21 bp were observed and 8 overlapping regions with 43 bp were observed as reported earlier (Yang et al. 2017).

Two non-protein coding ribosomal genes, 12s rDNA and 16s rDNA were of 942 bp and 1637 bp, respectively. Length of 22 tRNA genes ranged from 66 bp (tRNA-Cys) to 74 bp (tRNA-Leu). The WANCY region contained the 50 bp O_L_ light strand origin of replication, suggesting to play an important role in regulating light strand replication (Wong et al. 1983; Boore 1999). The control region (CR) or D-loop, the major non-coding region and commonly located between tRNA-Pro and tRNA-Phe genes, was 912 bp in length. As observed in other vertebrate mitogenomes, the CR of *M. deocata* contained similar conserved sequence blocks, including TAS, the central conserved sequence blocks (CSB-F, D, B and A) and conserved sequence blocks (CSB-2 and 3) (Wang et al. 2017).

**Figure 1.**
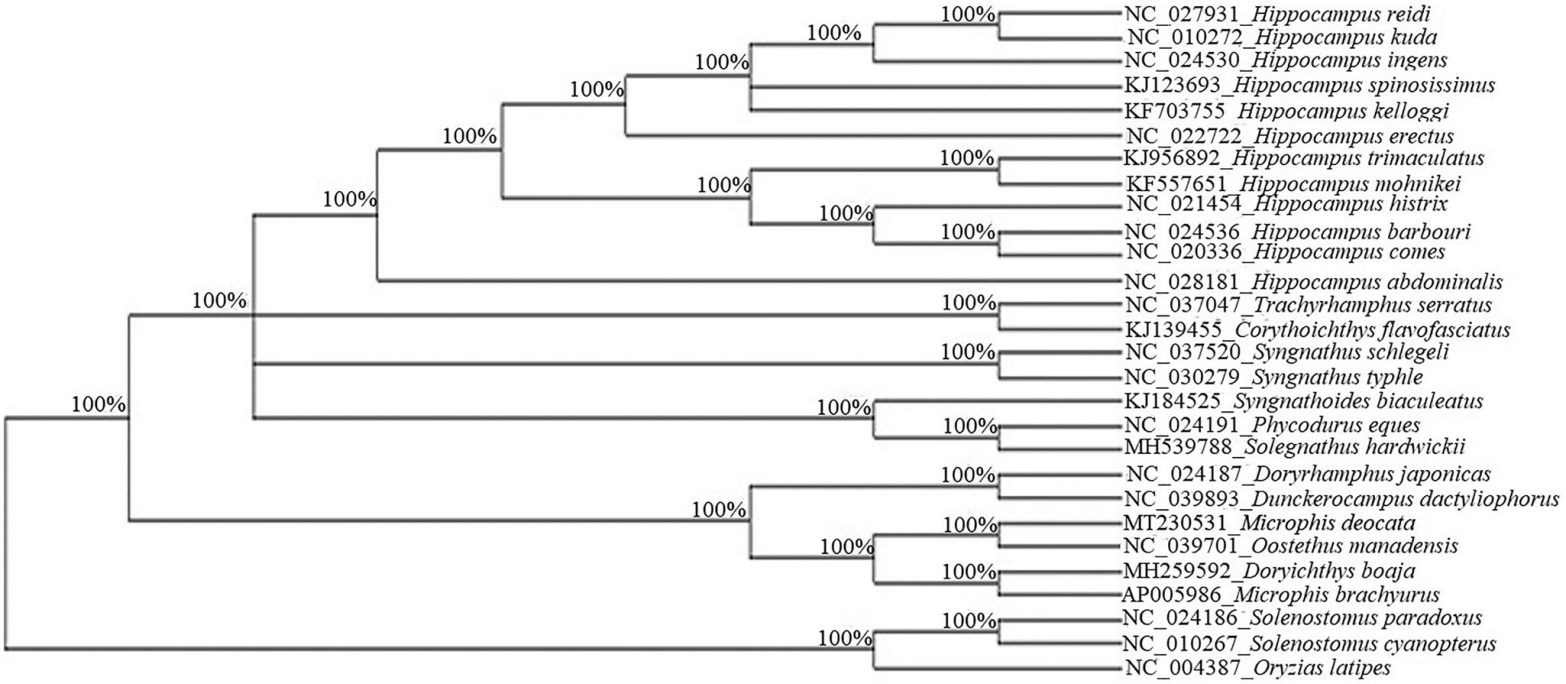
Phylogenetic tree of 27 Syngnathids based on 13 protein-coding genes.

Based on the oconcatenated supergene using 13 PGCs from 27 Syngnathidae species (12 genera), a maximum likelihood phylogenetic tree (Figure 1) was constructed by taking *Oryzias latipes* as an out group. In the present study, *M. deocata* clustered with *M. manadensis* (*Oostethus manadensis*), forming a sister group with *Doryrhamphus japonicas* and *Dunckerocampus dactyliophorus.* As observed by Wang et al. (2019), *M. brachyurus* clustered with *Doryichthys boaja*. The sequence resource generated in the present study would play an important role in population genetics and developing conservation strategy of this species.

## Data Availability

The data that support the findings of this study are openly available in the GenBank (Accession no. MT230531) at https://www.ncbi.nlm.nih.gov/nuccore/1866760761.
